# 2-(4-Bromo­phen­yl)-*N*-(2,6-dimethyl­phen­yl)acetamide

**DOI:** 10.1107/S1600536812034617

**Published:** 2012-08-11

**Authors:** Hoong-Kun Fun, Ching Kheng Quah, Prakash S. Nayak, B. Narayana, B. K. Sarojini

**Affiliations:** a X-ray Crystallography Unit, School of Physics, Universiti Sains Malaysia, 11800 USM, Penang, Malaysia; b Department of Studies in Chemistry, Mangalore University, Mangalagangotri 574 199, India; c Department of Chemistry, P. A. College of Engineering, Nadupadavu, Mangalore 574 153, India

## Abstract

In the title compound, C_16_H_16_BrNO, the dihedral angle between the benzene rings is 69.8 (2)°. In the crystal, N—H⋯O hydrogen bonds link the mol­ecules into *C*(4) chains propagating in [100]. Adjacent mol­ecules in the chains are also linked by C—H⋯O inter­actions which, along with the N—H⋯O hydrogen bonds, generate *R*_2_^1^(6) loops.

## Related literature

For general background to the title compound and for related structures, see: Fun *et al.* (2011*a* ▶,*b* ▶, 2012*a* ▶,*b* ▶). For hydrogen-bond motifs, see: Bernstein *et al.* (1995 ▶).
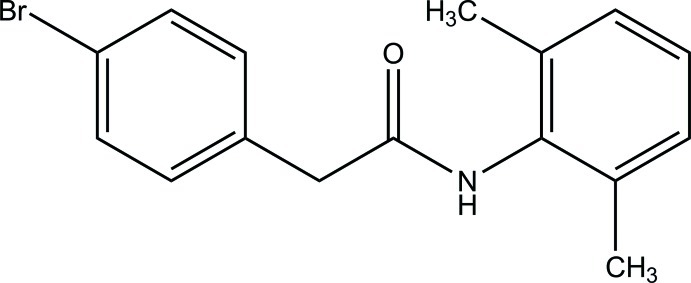


## Experimental

### 

#### Crystal data

C_16_H_16_BrNO
*M* *_r_* = 318.21
Monoclinic, 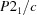

*a* = 4.7146 (6) Å
*b* = 22.999 (3) Å
*c* = 13.5350 (15) Å
β = 91.138 (3)°
*V* = 1467.3 (3) Å^3^
*Z* = 4
Mo *K*α radiation
μ = 2.79 mm^−1^
*T* = 296 K
0.18 × 0.09 × 0.07 mm

#### Data collection

Bruker SMART APEXII DUO CCD diffractometer
Absorption correction: multi-scan (*SADABS*; Bruker, 2009 ▶) *T*_min_ = 0.635, *T*_max_ = 0.822
14048 measured reflections
3352 independent reflections
1593 reflections with *I* > 2σ(*I*)
*R*_int_ = 0.074

#### Refinement

*R*[*F* ^2^ > 2σ(*F* ^2^)] = 0.052
*wR*(*F* ^2^) = 0.143
*S* = 1.01
3352 reflections
178 parameters
H atoms treated by a mixture of independent and constrained refinement
Δρ_max_ = 0.37 e Å^−3^
Δρ_min_ = −0.57 e Å^−3^

### 

Data collection: *APEX2* (Bruker, 2009 ▶); cell refinement: *SAINT* (Bruker, 2009 ▶); data reduction: *SAINT*; program(s) used to solve structure: *SHELXTL* (Sheldrick, 2008 ▶); program(s) used to refine structure: *SHELXTL*; molecular graphics: *SHELXTL*; software used to prepare material for publication: *SHELXTL* and *PLATON* (Spek, 2009 ▶).

## Supplementary Material

Crystal structure: contains datablock(s) global, I. DOI: 10.1107/S1600536812034617/hb6926sup1.cif

Structure factors: contains datablock(s) I. DOI: 10.1107/S1600536812034617/hb6926Isup2.hkl

Supplementary material file. DOI: 10.1107/S1600536812034617/hb6926Isup3.cml

Additional supplementary materials:  crystallographic information; 3D view; checkCIF report

## Figures and Tables

**Table 1:** Hydrogen-bond geometry (Å, °)

*D*—H⋯*A*	*D*—H	H⋯*A*	*D*⋯*A*	*D*—H⋯*A*
N1—H1*N*1⋯O1^i^	0.91 (4)	1.95 (4)	2.847 (4)	166 (3)
C7—H7*B*⋯O1^i^	0.97	2.38	3.240 (5)	148

Symmetry code: (i) 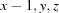.

**Fig. 1.**
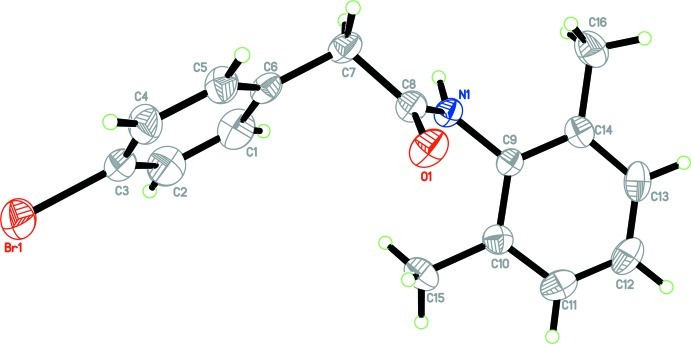
The molecular structure of the title compound showing 30% probability displacement ellipsoids for non-H atoms.

**Fig. 2.**
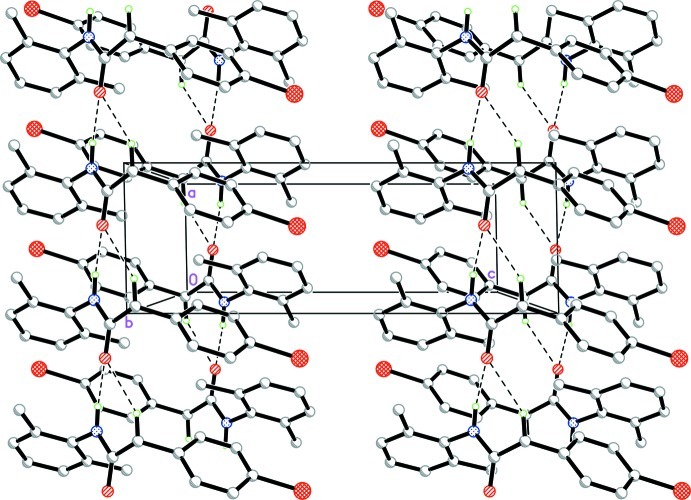
The crystal structure of the title compound, viewed along the b axis. H atoms not involved in hydrogen bonds (dashed lines) have been omitted for clarity.

## supplementary crystallographic information

### Comment

In continuation of our work on synthesis of amides (Fun *et al.*, 2011*a*, 2011*b*, 2012*a*, 2012*b*), we report herein the crystal structure of the title compound.

In the title molecule (Fig. 1), the two benzene rings (C1-C6 and C9-C14) make a dihedral angle of 69.8 (2)°. Bond lengths and angles are within normal ranges and are comparable to related structures (Fun *et al.*, 2011*a*, 2011*b*, 2012*a*, 2012*b*).

In the crystal structure, Fig. 2, molecules are linked *via* N1–H1N1···O1 and C7–H7B···O1 hydrogen bonds (Table 1) into one-dimensional [100] chains which contain R_2_^1^ (6) ring motifs (Bernstein *et al.*, 1995).

### Experimental

4-Bromophenylacetic acid (0.213 g, 1 mmol), 2,6-Dimethylaniline (0.1 ml, 1 mmol) and 1-ethyl-3-(3-dimethylaminopropyl)-carbodiimide hydrochloride (1.0 g, 0.01 mol) were dissolved in dichloromethane (20 ml). The mixture was stirred in presence of triethylamine at 273 K for about 3 h. The contents were poured into 100 ml of ice-cold aqueous hydrochloric acid with stirring. The resulting solution was extracted thrice with dichloromethane. The organic layer was washed with saturated NaHCO_3_ solution and brine solution, dried and concentrated under reduced pressure to give the title compound (I). Colourless plates were grown from a methanol and *N,N*-dimethylformamide (1:1) solvent mixture by the slow evaporation method (*m.p.*:479-481 K).

### Refinement

Atom H1N1 was located in a difference Fourier map and refined freely [N–H = 0.91 (4) Å]. The remaining H atoms were positioned geometrically and refined using a riding model with C–H = 0.93-0.97 Å and *U*_iso_(H) = 1.2 or 1.5 *U*_eq_(C). A rotating-group model was applied for the methyl groups.

### Crystal data

**Table:**

C_16_H_16_BrNO	*F*(000) = 648
*M**_r_* = 318.21	*D*_x_ = 1.440 Mg m^−^^3^
Monoclinic, *P*2_1_/*c*	Mo *K*α radiation, λ = 0.71073 Å
Hall symbol: -P 2ybc	Cell parameters from 1376 reflections
*a* = 4.7146 (6) Å	θ = 3.0–20.1°
*b* = 22.999 (3) Å	µ = 2.79 mm^−^^1^
*c* = 13.5350 (15) Å	*T* = 296 K
β = 91.138 (3)°	Plate, colourless
*V* = 1467.3 (3) Å^3^	0.18 × 0.09 × 0.07 mm
*Z* = 4	

### Data collection

**Table:**

Bruker SMART APEXII DUO CCD diffractometer	3352 independent reflections
Radiation source: fine-focus sealed tube	1593 reflections with *I* > 2σ(*I*)
Graphite monochromator	*R*_int_ = 0.074
φ and ω scans	θ_max_ = 27.5°, θ_min_ = 1.8°
Absorption correction: multi-scan (*SADABS*; Bruker, 2009)	*h* = −6→6
*T*_min_ = 0.635, *T*_max_ = 0.822	*k* = −26→29
14048 measured reflections	*l* = −17→17

### Refinement

**Table:**

Refinement on *F*^2^	Primary atom site location: structure-invariant direct methods
Least-squares matrix: full	Secondary atom site location: difference Fourier map
*R*[*F*^2^ > 2σ(*F*^2^)] = 0.052	Hydrogen site location: inferred from neighbouring sites
*wR*(*F*^2^) = 0.143	H atoms treated by a mixture of independent and constrained refinement
*S* = 1.01	*w* = 1/[σ^2^(*F*_o_^2^) + (0.0518*P*)^2^ + 0.539*P*] where *P* = (*F*_o_^2^ + 2*F*_c_^2^)/3
3352 reflections	(Δ/σ)_max_ = 0.001
178 parameters	Δρ_max_ = 0.37 e Å^−^^3^
0 restraints	Δρ_min_ = −0.57 e Å^−^^3^

### Special details

**Table:**

Geometry. All esds (except the esd in the dihedral angle between two l.s. planes) are estimated using the full covariance matrix. The cell esds are taken into account individually in the estimation of esds in distances, angles and torsion angles; correlations between esds in cell parameters are only used when they are defined by crystal symmetry. An approximate (isotropic) treatment of cell esds is used for estimating esds involving l.s. planes.
Refinement. Refinement of F^2^ against ALL reflections. The weighted R-factor wR and goodness of fit S are based on F^2^, conventional R-factors R are based on F, with F set to zero for negative F^2^. The threshold expression of F^2^ > 2sigma(F^2^) is used only for calculating R-factors(gt) etc. and is not relevant to the choice of reflections for refinement. R-factors based on F^2^ are statistically about twice as large as those based on F, and R- factors based on ALL data will be even larger.

### Fractional atomic coordinates and isotropic or equivalent isotropic displacement parameters (Å^2^)

**Table:**

	*x*	*y*	*z*	*U*_iso_*/*U*_eq_	
Br1	0.41005 (16)	0.35233 (3)	0.61576 (4)	0.1038 (3)	
O1	0.4016 (5)	0.33510 (13)	1.1247 (2)	0.0615 (8)	
N1	−0.0290 (7)	0.37234 (13)	1.1589 (2)	0.0416 (7)	
C1	0.0074 (10)	0.35773 (19)	0.8830 (4)	0.0688 (13)	
H1A	−0.1203	0.3825	0.9134	0.083*	
C2	0.0970 (11)	0.3706 (2)	0.7894 (4)	0.0744 (14)	
H2A	0.0300	0.4037	0.7570	0.089*	
C3	0.2837 (10)	0.33480 (19)	0.7448 (3)	0.0614 (11)	
C4	0.3828 (10)	0.28624 (19)	0.7918 (3)	0.0652 (12)	
H4A	0.5100	0.2616	0.7609	0.078*	
C5	0.2916 (9)	0.27413 (17)	0.8861 (3)	0.0550 (10)	
H5A	0.3608	0.2413	0.9186	0.066*	
C6	0.1019 (8)	0.30930 (17)	0.9327 (3)	0.0469 (9)	
C7	0.0048 (8)	0.29567 (17)	1.0356 (3)	0.0531 (10)	
H7A	0.0515	0.2557	1.0517	0.064*	
H7B	−0.1996	0.3000	1.0383	0.064*	
C8	0.1443 (8)	0.33567 (16)	1.1105 (3)	0.0464 (9)	
C9	0.0737 (7)	0.41182 (15)	1.2329 (2)	0.0380 (8)	
C10	0.2465 (8)	0.45805 (16)	1.2062 (3)	0.0477 (9)	
C11	0.3468 (10)	0.49491 (18)	1.2805 (3)	0.0664 (12)	
H11A	0.4670	0.5254	1.2645	0.080*	
C12	0.2719 (10)	0.4871 (2)	1.3765 (3)	0.0704 (13)	
H12A	0.3412	0.5122	1.4251	0.084*	
C13	0.0963 (10)	0.4428 (2)	1.4010 (3)	0.0618 (12)	
H13A	0.0450	0.4383	1.4666	0.074*	
C14	−0.0091 (8)	0.40372 (16)	1.3301 (3)	0.0470 (9)	
C15	0.3245 (11)	0.4697 (2)	1.1008 (3)	0.0736 (13)	
H15A	0.1636	0.4621	1.0581	0.110*	
H15B	0.4787	0.4448	1.0829	0.110*	
H15C	0.3808	0.5096	1.0941	0.110*	
C16	−0.2007 (10)	0.35451 (19)	1.3576 (3)	0.0680 (12)	
H16A	−0.1351	0.3192	1.3278	0.102*	
H16B	−0.3903	0.3627	1.3344	0.102*	
H16C	−0.1992	0.3502	1.4281	0.102*	
H1N1	−0.220 (8)	0.3663 (14)	1.153 (3)	0.042 (10)*	

### Atomic displacement parameters (Å^2^)

**Table:**

	*U*^11^	*U*^22^	*U*^33^	*U*^12^	*U*^13^	*U*^23^
Br1	0.1668 (7)	0.0908 (4)	0.0537 (3)	−0.0423 (4)	0.0020 (3)	0.0035 (3)
O1	0.0288 (15)	0.081 (2)	0.0748 (19)	0.0040 (14)	0.0040 (14)	−0.0234 (15)
N1	0.0260 (17)	0.0529 (19)	0.0457 (17)	0.0002 (15)	−0.0001 (15)	−0.0070 (14)
C1	0.063 (3)	0.064 (3)	0.079 (3)	0.022 (2)	−0.002 (3)	−0.012 (3)
C2	0.085 (4)	0.061 (3)	0.076 (3)	0.013 (3)	−0.014 (3)	0.008 (2)
C3	0.077 (3)	0.055 (3)	0.052 (2)	−0.015 (2)	−0.003 (2)	−0.005 (2)
C4	0.083 (3)	0.056 (3)	0.057 (3)	0.007 (2)	0.015 (3)	−0.011 (2)
C5	0.064 (3)	0.047 (2)	0.053 (2)	0.010 (2)	0.005 (2)	−0.0060 (18)
C6	0.038 (2)	0.050 (2)	0.053 (2)	−0.0018 (19)	−0.0050 (19)	−0.0126 (19)
C7	0.036 (2)	0.062 (3)	0.061 (2)	−0.0096 (19)	0.010 (2)	−0.021 (2)
C8	0.033 (2)	0.057 (2)	0.050 (2)	0.0006 (19)	0.0076 (19)	−0.0070 (18)
C9	0.0304 (19)	0.041 (2)	0.0421 (19)	0.0044 (16)	−0.0021 (17)	−0.0023 (16)
C10	0.047 (2)	0.044 (2)	0.052 (2)	0.0030 (19)	−0.0013 (19)	0.0040 (18)
C11	0.067 (3)	0.052 (3)	0.079 (3)	−0.009 (2)	−0.005 (3)	−0.010 (2)
C12	0.079 (3)	0.066 (3)	0.066 (3)	0.000 (3)	−0.011 (3)	−0.024 (2)
C13	0.071 (3)	0.074 (3)	0.040 (2)	0.016 (3)	0.000 (2)	−0.010 (2)
C14	0.042 (2)	0.052 (2)	0.048 (2)	0.0086 (19)	0.0069 (19)	0.0016 (18)
C15	0.090 (4)	0.069 (3)	0.062 (3)	−0.014 (3)	0.006 (3)	0.019 (2)
C16	0.061 (3)	0.084 (3)	0.059 (3)	0.002 (3)	0.017 (2)	0.010 (2)

### Geometric parameters (Å, º)

**Table:**

Br1—C3	1.899 (4)	C7—H7B	0.9700
O1—C8	1.224 (4)	C9—C10	1.392 (5)
N1—C8	1.353 (5)	C9—C14	1.392 (5)
N1—C9	1.429 (4)	C10—C11	1.391 (5)
N1—H1N1	0.91 (4)	C10—C15	1.504 (5)
C1—C6	1.371 (6)	C11—C12	1.365 (6)
C1—C2	1.376 (6)	C11—H11A	0.9300
C1—H1A	0.9300	C12—C13	1.358 (6)
C2—C3	1.356 (6)	C12—H12A	0.9300
C2—H2A	0.9300	C13—C14	1.400 (5)
C3—C4	1.363 (6)	C13—H13A	0.9300
C4—C5	1.383 (5)	C14—C16	1.500 (5)
C4—H4A	0.9300	C15—H15A	0.9600
C5—C6	1.369 (5)	C15—H15B	0.9600
C5—H5A	0.9300	C15—H15C	0.9600
C6—C7	1.508 (5)	C16—H16A	0.9600
C7—C8	1.510 (5)	C16—H16B	0.9600
C7—H7A	0.9700	C16—H16C	0.9600
			
C8—N1—C9	122.4 (3)	C10—C9—C14	121.6 (3)
C8—N1—H1N1	118 (2)	C10—C9—N1	119.7 (3)
C9—N1—H1N1	118 (2)	C14—C9—N1	118.7 (3)
C6—C1—C2	121.6 (4)	C11—C10—C9	118.0 (4)
C6—C1—H1A	119.2	C11—C10—C15	119.4 (4)
C2—C1—H1A	119.2	C9—C10—C15	122.6 (4)
C3—C2—C1	119.5 (4)	C12—C11—C10	121.2 (4)
C3—C2—H2A	120.2	C12—C11—H11A	119.4
C1—C2—H2A	120.2	C10—C11—H11A	119.4
C2—C3—C4	120.6 (4)	C13—C12—C11	120.2 (4)
C2—C3—Br1	119.9 (4)	C13—C12—H12A	119.9
C4—C3—Br1	119.5 (3)	C11—C12—H12A	119.9
C3—C4—C5	119.1 (4)	C12—C13—C14	121.6 (4)
C3—C4—H4A	120.5	C12—C13—H13A	119.2
C5—C4—H4A	120.5	C14—C13—H13A	119.2
C6—C5—C4	121.6 (4)	C9—C14—C13	117.4 (4)
C6—C5—H5A	119.2	C9—C14—C16	121.2 (3)
C4—C5—H5A	119.2	C13—C14—C16	121.4 (4)
C5—C6—C1	117.6 (4)	C10—C15—H15A	109.5
C5—C6—C7	121.0 (4)	C10—C15—H15B	109.5
C1—C6—C7	121.3 (4)	H15A—C15—H15B	109.5
C6—C7—C8	110.9 (3)	C10—C15—H15C	109.5
C6—C7—H7A	109.5	H15A—C15—H15C	109.5
C8—C7—H7A	109.5	H15B—C15—H15C	109.5
C6—C7—H7B	109.5	C14—C16—H16A	109.5
C8—C7—H7B	109.5	C14—C16—H16B	109.5
H7A—C7—H7B	108.0	H16A—C16—H16B	109.5
O1—C8—N1	122.4 (3)	C14—C16—H16C	109.5
O1—C8—C7	121.1 (3)	H16A—C16—H16C	109.5
N1—C8—C7	116.5 (3)	H16B—C16—H16C	109.5
			
C6—C1—C2—C3	−0.1 (7)	C8—N1—C9—C10	67.8 (5)
C1—C2—C3—C4	0.1 (7)	C8—N1—C9—C14	−113.9 (4)
C1—C2—C3—Br1	−179.7 (3)	C14—C9—C10—C11	3.0 (5)
C2—C3—C4—C5	−0.4 (7)	N1—C9—C10—C11	−178.8 (3)
Br1—C3—C4—C5	179.4 (3)	C14—C9—C10—C15	−176.3 (4)
C3—C4—C5—C6	0.8 (7)	N1—C9—C10—C15	2.0 (5)
C4—C5—C6—C1	−0.9 (6)	C9—C10—C11—C12	−1.8 (6)
C4—C5—C6—C7	−179.8 (4)	C15—C10—C11—C12	177.4 (4)
C2—C1—C6—C5	0.5 (6)	C10—C11—C12—C13	0.0 (7)
C2—C1—C6—C7	179.5 (4)	C11—C12—C13—C14	0.9 (7)
C5—C6—C7—C8	104.8 (4)	C10—C9—C14—C13	−2.2 (5)
C1—C6—C7—C8	−74.1 (5)	N1—C9—C14—C13	179.6 (3)
C9—N1—C8—O1	−2.2 (6)	C10—C9—C14—C16	178.6 (4)
C9—N1—C8—C7	178.9 (3)	N1—C9—C14—C16	0.3 (5)
C6—C7—C8—O1	−63.7 (5)	C12—C13—C14—C9	0.2 (6)
C6—C7—C8—N1	115.2 (4)	C12—C13—C14—C16	179.4 (4)

### Hydrogen-bond geometry (Å, º)

**Table:**

*D*—H···*A*	*D*—H	H···*A*	*D*···*A*	*D*—H···*A*
N1—H1*N*1···O1^i^	0.91 (4)	1.95 (4)	2.847 (4)	166 (3)
C7—H7*B*···O1^i^	0.97	2.38	3.240 (5)	148

Symmetry code: (i) *x*−1, *y*, *z*.

## References

[bb1] Bernstein, J., Davis, R. E., Shimoni, L. & Chang, N.-L. (1995). *Angew. Chem. Int. Ed. Engl.* **34**, 1555–1573.

[bb2] Bruker (2009). *APEX2*, *SAINT* and *SADABS* Bruker AXS Inc., Madison, Wisconsin, USA.

[bb3] Fun, H.-K., Quah, C. K., Narayana, B., Nayak, P. S. & Sarojini, B. K. (2011*a*). *Acta Cryst.* E**67**, o2926–o2927. 10.1107/S1600536811041110 PMC3247340 22219958

[bb4] Fun, H.-K., Quah, C. K., Narayana, B., Nayak, P. S. & Sarojini, B. K. (2011*b*). *Acta Cryst.* E**67**, o2941–o2942. 10.1107/S1600536811041468 PMC3247353 22219971

[bb5] Fun, H.-K., Quah, C. K., Nayak, P. S., Narayana, B. & Sarojini, B. K. (2012*a*). *Acta Cryst.* E**68**, o1385. 10.1107/S1600536812014869 PMC3344513 22590275

[bb6] Fun, H.-K., Quah, C. K., Nayak, P. S., Narayana, B. & Sarojini, B. K. (2012*b*). *Acta Cryst.* E**68**, o2461. 10.1107/S1600536812031595 PMC3414917 22904904

[bb7] Sheldrick, G. M. (2008). *Acta Cryst.* A**64**, 112–122. 10.1107/S0108767307043930 18156677

[bb8] Spek, A. L. (2009). *Acta Cryst.* D**65**, 148–155. 10.1107/S090744490804362X PMC2631630 19171970

